# Structural basis for decreased induction of class IB PI3‐kinases expression by MIF inhibitors

**DOI:** 10.1111/jcmm.12949

**Published:** 2016-09-13

**Authors:** Abhay Kumar Singh, Georgios Pantouris, Sebastian Borosch, Siripong Rojanasthien, Thomas Yoonsang Cho

**Affiliations:** ^1^ Edward A. Doisy Department of Biochemistry and Molecular BiologySaint Louis University School of MedicineSt. LouisMOUSA; ^2^ Department of PharmacologyYale University School of MedicineNew HavenCTUSA; ^3^ Institute of Biochemistry and Molecular Cell BiologyRWTH Aachen UniversityAachenGermany

**Keywords:** MIF, Class IB PI3K, p101, p110 gamma

## Abstract

Macrophage migration inhibitory factor (MIF) is a master regulator of proinflammatory cytokines and plays pathological roles when not properly regulated in rheumatoid arthritis, lupus, atherosclerosis, asthma and cancer. Unlike canonical cytokines, MIF has vestigial keto‐enol tautomerase activity. Most of the current MIF inhibitors were screened for the inhibition of this enzymatic activity. However, only some of the enzymatic inhibitors inhibit receptor‐mediated biological functions of MIF, such as cell recruitment, through an unknown molecular mechanism. The goal of this study was to understand the molecular basis underlying the pharmacological inhibition of biological functions of MIF. Here, we demonstrate how the structural changes caused upon inhibitor binding translate into the alteration of MIF‐induced downstream signalling. Macrophage migration inhibitory factor activates phosphoinositide 3‐kinases (PI3Ks) that play a pivotal role in immune cell recruitment in health and disease. There are several different PI3K isoforms, but little is known about how they respond to MIF. We demonstrate that MIF up‐regulates the expression of Class IB PI3Ks in leucocytes. We also demonstrate that MIF tautomerase active site inhibitors down‐regulate the expression of Class IB PI3Ks as well as leucocyte recruitment *in vitro* and *in vivo*. Finally, based on our MIF:inhibitor complex crystal structures, we hypothesize that the reduction in Class IB PI3K expression occurs because of the displacement of Pro1 towards the second loop of MIF upon inhibitor binding, which results in increased flexibility of the loop 2 and sub‐optimal MIF binding to its receptors. These results will provide molecular insights for fine‐tuning the biological functions of MIF.

## Introduction

Macrophage migration inhibitory factor (MIF) is expressed at high levels in various inflammatory diseases and cancer 1. Macrophage migration inhibitory factor promotes directional cell migration (or chemotaxis) of various cell types 2, 3, 4. The mechanism underlying chemotaxis entails MIF activation of phosphoinositide 3‐kinases (PI3Ks) that are upstream activators of the pleckstrin homology domain‐containing protein kinase B (AKT) pathway 5. Macrophage migration inhibitory factor triggers the PI3K/AKT signalling pathway by binding to its cell surface receptor CD74 in complex with CD44 as well as recently identified C‐X‐C chemokine receptors, CXCR2, CXCR4 and CXCR7 6, 7. A proposed mechanism for the MIF activation of PI3Ks involves G_α_ and G_βγ_ proteins dissociated from the activated receptors 8.

There are three classes in the PI3K family 8. Among those, Class I PI3Ks are responsible for immune cell recruitment 9. Class I PI3K is composed of a heterodimer of a regulatory subunit (p85 or p101) and a catalytic subunit (p110α, β, δ or γ). The catalytic subunits, α, β and δ are regulated by p85 (Class IA) 10, 11, whereas the γ subunit is regulated by p101 (Class IB) 12. In neutrophils, chemokines can activate all these catalytic subunits 5, 13. P110δ 14, 15 and γ 9, 16, 17 contribute to neutrophil chemotaxis. In particular, maximal activation of p110γ is associated with p101 12, 18, whereas other catalytic subunits are negatively regulated by p85 19, 20. However, it is controversial as to whether Class IA or Class IB is the major isoform involved in the production of phosphatidylinositol (3,4,5)‐trisphosphate and the activation of downstream signalling pathways 21, 22.

Unlike canonical cytokines and chemokines, a trimeric MIF molecule has three tautomerase active sites at the trimer interfaces 23. This active site has been a screening target for small molecule inhibitors in an effort to develop anti‐MIF drugs. Although the enzymatic activity is vestigial, there is accumulating evidence that the active site is important for receptor binding 24, 25. We recently found that the residues located at the rim of the active site are important for neutrophil recruitment through a mutational study 26. We also found that the rigidity of the active site residues is necessary for MIF‐induced neutrophil recruitment to the lungs. However, a systematic correlation study between the active site residues potential to interact with receptors and the activation of downstream signalling molecules has not been performed.

In this study, we report that MIF increases the expression levels of Class IB PI3Ks, leading to leucocyte chemotaxis. The expression levels of Class IB PI3Ks are down‐regulated when MIF is occupied with active site inhibitors that induce the displacement of Pro1 towards the second loop (L2) of MIF. Together, correlating the inhibitor‐induced structural alteration of the MIF active site with the changed MIF activation of downstream signalling molecules contributes to identifying biologically relevant structural determinants of MIF.

## Materials and methods

### Materials

4‐Hydroxyphenylpyruvic acid (HPP), **1** and **12** were purchased from Sigma‐Aldrich (St. Louis, MO, USA). **2** was purchased from Maybridge (part of Thermo Fisher Scientific, Waltham, MA, USA). THP‐1 cells (monocytic cell line) were purchased from American Type Culture Collection. HL60 (human promyelocytic leukaemia cell line) cells were a kind gift from Dr. David Ford (Dept. of Biochemistry and Molecular Biology, Saint Louis University). Recombinant human MIF was expressed in BL21(DE3) *Escherichia coli* cells and enriched in flow‐through by interconnected anion‐ (Diethylaminoethyl Sepharose Fast Flow) and cation‐ (SP Sepharose Fast Flow) exchange columns (GE Healthcare Life Sciences, Pittsburgh, PA, USA). Macrophage migration inhibitory factor in the flow‐through was further purified by Superdex 200 size exclusion column (>95% purity based on Coomassie staining). For cell‐based assays, the column‐purified protein was run through a Sep‐Pak reverse‐phase column from Waters (Milford, MA, USA) in a denaturing condition (e.g. acetonitrile gradient) to remove endotoxin. Resulting endotoxin‐free MIF was refolded in a phosphate buffer at pH 7.4. Endotoxin level was <0.1 EU/ml when measured using a Kinetic Turbidimetric LAL reagent from Charles River (Skokie, IL, USA).

### PI3K signalling assay

THP‐1 and HL60 cells were starved overnight in RPMI 1640 medium with 0.5% heat‐inactivated fetal bovine serum (FBS). The cells were then treated with MIF for 3 hrs. To evaluate the MIF inhibitors, 50 ng/ml of MIF was incubated with 0.5 μM of inhibitors for 2 hrs at room temperature prior to the treatment of THP‐1 and HL60 cells. After the incubation, the cells were lysed in 2× SDS loading buffer by sonication and subjected to Western blot analysis. The blots were probed with anti‐phospho‐PI3Kp85 (Tyr458)/p55 (Tyr199) (Specificity: mouse [and species predicted to react based on 100% sequence homology: human, rat, monkey, bovine], species of origin: mouse, source: rabbit, Cat#4228, lot#2), anti‐PI3Kp85 (19H8) (Specificity: human, mouse, rat, species of origin: human, source: rabbit, Cat#4257, lot#6), anti‐PI3Kp110α (C73F8) (Specificity: human, mouse, rat, bovine, species of origin: human, source: rabbit, Cat#4249, lot#7), anti‐PI3Kp110β (C33D4) (Specificity: human, species of origin: human, source: rabbit, Cat#3011, lot#6), anti‐PI3Kp110γ (D55D5) (Specificity: human, mouse, species of origin: human, source: rabbit, Cat#5405, lot#4) and anti‐PI3Kp101 (5569S) (Specificity: human, mouse, species of origin: human, source: rabbit, Cat#5569, lot#1) antibodies from Cell Signaling Technology (Danvers, MA, USA). To confirm equal loading, membranes were stripped and re‐probed with α‐tubulin (Sigma‐Aldrich). The blots were developed using a chemiluminescence detection system (Thermo Fisher Scientific). Bands were quantified by performing densitometric analysis using ImageJ software. All assays reported here were repeated at least three times independently with three replicates per assay unless otherwise stated.

### Time‐dependent HPP tautomerase inhibition assay

Covalent inhibitor binding to MIF was examined as described earlier 14, 27. Briefly, each inhibitor (1 mM) was incubated with MIF (20 μM) in 20 mM Tris, pH7.4 and 20 mM NaCl at room temperature. An aliquot of the incubation mixture (1.5 μl) was removed and added to a MIF premix for HPP tautomerase assay at final concentrations of 10 μM inhibitor and 200 nM MIF as described 9, 28.

### Cell chemotaxis inhibition assay

Inhibition of THP‐1 and HL60 cell migration was studied using the ChemoTx 96‐well disposable chamber with a framed filter with 5 μm pores (Neuroprobe, Inc., Gaithersburg, MD, USA) and quantitated using the AQueous cell proliferation assay kit (Promega, Madison, WI, USA). Cells were harvested and re‐suspended in the assay medium (RPMI 1640 medium with 0.5% FBS, 1× Pen Strep) at the density of 10^6^ cells/mL. Macrophage migration inhibitory factor (50 ng/ml) was incubated with each inhibitor (0.5 μM) at room temperature for 1 hr. After the incubation, 30 μl of each MIF/inhibitor mixture was transferred to the bottom of the chemotaxis chamber and 50 μl of cells was transferred to the top of the chemotaxis chamber. As quantitation standards for migrated cells, a separate set of cells diluted in a series were transferred to the lower chambers within one section of the assay plate without addition of MIF inhibitor mixture. Then, the assay plate was incubated for 150 min. at 37°C. Cells remained on the top of the filter were removed using a cell harvester and were flushed with phosphate‐buffered saline. The plate was centrifuged at 500 × g for 10 min. to settle all migrated cells down to the bottom chamber. The migrated cells (5 μl) were mixed with the same volume of the CellTiter 96^®^ AQueous One Solution Reagent (Promega) and incubated for 3 hrs at 37°C. Then, formazan formation was measured using an Enspire multimode plate reader (PerkinElmer, Waltham, MA, USA) at 492 nm. Results were presented as a migration index. Each assay was carried out in triplicate. We conducted Student's *t*‐test with the migration indexes to compare cells treated and untreated with inhibitors.

### Flow cytometry

Surface staining of CXCR2, CXCR4, CXCR7 and CD74 was performed on THP‐1 and HL60 cells. Cells were collected from plates using a cell scraper. Then, cells (0.1 × 10^6^) were washed with PBS and re‐suspended in a FACS staining buffer (PBS, 0.1% sodium azide, 5% FBS). Nonspecific binding of antibodies to Fc receptors was blocked with Fc blocker (BD Biosciences, San Jose, CA, USA). Cells were incubated with fluorochrome‐conjugated antibodies (CXCR2‐PE, CXCR4‐APC, CXCR7‐PerCP and CD74‐FITC) and their receptive isotype controls for 1 hr at 4°C. All antibodies were from eBioscience (San Diego, CA, USA). The cells were then washed with FACS staining buffer and tested immediately using a FACS LSRII flow cytometer (BD Biosciences) at the Flow Cytometry Research Core at Saint Louis University. Analysis was performed with Flow Jo software (Treestar Inc, Ashland, OR).

### X‐ray crystallography

Column‐purified MIF was concentrated to 10 mg/ml and crystallized in complex with 2.5 mM of each inhibitor in 0.1 M TrisHCl, pH 7.5, 1.9 M (NH_4_)_2_SO_4_, and 3% isopropanol. Crystals were grown at 18°C within 2 weeks. X‐ray diffraction data were collected from a single crystal for each inhibitor at X29 beamline of National Synchrotron Light Source at the Brookhaven National Laboratory. The diffraction data were indexed using MOSFLM 29 and reduced using SCALA in the CCP4 program package 30. Molecular replacement was performed with PHASER 31 with a previously determined MIF structure (PDB ID: 1MIF) as a search template. Visual inspection and manual refinement of structural models were performed with COOT 32. Computational refinement between manual refinements was performed by REFMAC 33.

### B‐factor analysis

A high‐resolution wild‐type apo‐MIF crystal structure (PDB ID: 3DJH) was used as the reference for structural comparison with inhibitor‐bound ones. B‐factor values, indicating the degree of flexibility, for backbone alpha carbon (CA) atoms were obtained from PDB files for the apo‐ and the inhibitor‐bound MIF structures and graphed using Prism6. For structural comparison, all the inhibitor‐bound structures were loaded into PyMOL software and aligned to the apo‐structure. The built‐in Putty function in PyMOL was used for the graphical presentation of B‐factor.

### *In vivo* neutrophil recruitment assay

Neutrophil recruitment assay was performed in the lungs of male C57BL/6J mice (8–12 weeks old) as described 34. Briefly, 1 mg of either MIF or MIF pre‐incubated with covalent inhibitors, at 1:1 stoichiometric ratio, was administrated to murine lungs (three mice per inhibitor) *via* the intranasal route in 50 ml of saline solution. After 5 hrs, the mice were killed and the BAL fluids were collected (3 × 0.8 ml PBS) and transferred to labelled microscope slides for analysis. After an overnight incubation at room temperature, the dried slides were stained using HEMA 3 (Thermo Fisher Scientific, St. Louis, MO, USA). The percentage of recruited neutrophils was calculated based on the count of minimum 200 cells. Statistical analysis was carried out using the two‐tailed t‐test. Each experiment was repeated in triplicate. The animal protocol was approved by the Yale University Institutional Animal Care and Use Committee (Approval# 2014‐11570).

### PI/Annexin‐V cell viability assay

The frequency of apoptosis in THP‐1 and HL60 cells following treatment with MIF inhibitors (1, 2, 12 and ISO‐1) was assayed. Cells were treated with MIF (50 ng/ml) and its inhibitors (0.5 μM) for 180 min. After termination of the culture, cells were washed twice with PBS and re‐suspended in 100 μl of Annexin‐V binding buffer (Cat#556547; BD Biosciences). To the cell suspension, 5 μl of FITC‐conjugated Annexin‐V and 10 μl of PI (50 μg/ml) solution were added and further incubated for 15 min. at room temperature. After staining, 400 μl of Annexin binding buffer was added, and the samples were stored on ice until data acquisition. About 10,000 events were acquired in FACS Canto flow cytometer (BD Biosciences). The results were represented as dot plots of FITC *versus* PI. The frequency of cells in each quadrant, Q1 (FITC‐/PI+, dead), Q2 (FITC+/PI+, late apoptosis), Q3 (FITC+/PI−, early apoptosis) and Q4 (FITC−/PI−, healthy) was determined using Flow Jo software (FlowJo LLC., Ashland, OR, USA).

## Results

### MIF activates the expression of p110γ and p101 subunits of the Class IB PI3K

**Figure 1:**
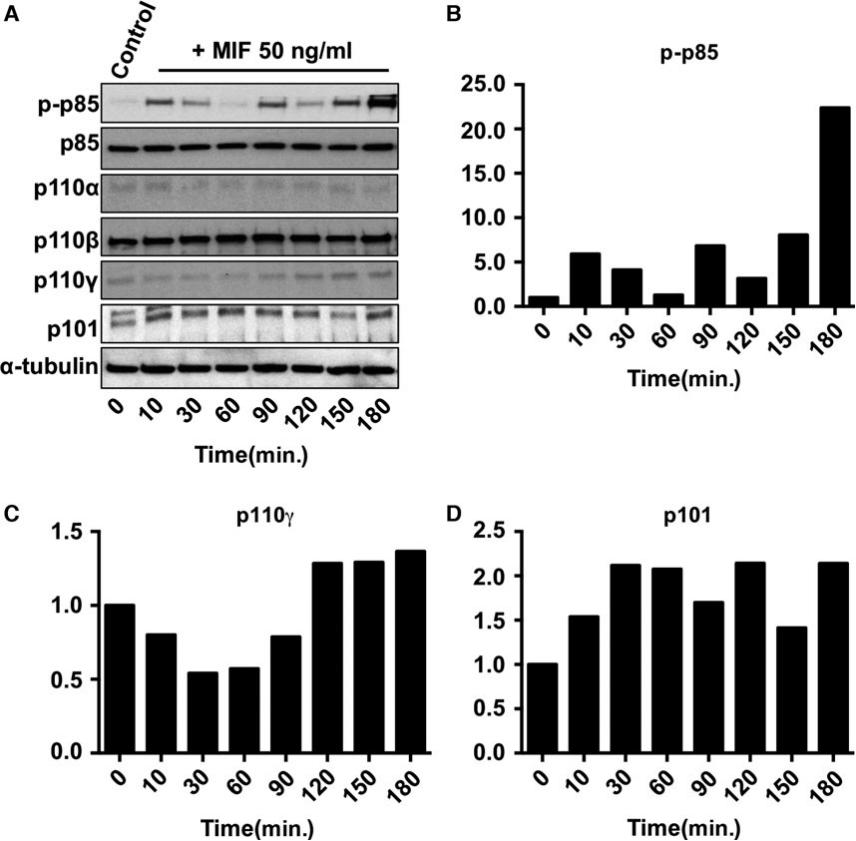
MIF activation of Class IB PI3Ks expression in THP‐1 monocytes. (**A**) Western blots show the expression of phosphorylated‐p85, total p‐85, p110α, p110β, p110γ and p101 monitored from 10 to 180 min after the MIF activation of THP‐1 cells. All controls were measured at *t* = 0 without MIF activation. (**B**–**D**) Bar diagrams show fold changes in intensity for p‐p85, p110γ and p101 respectively. The intensity data were analysed by ImageJ software and normalized to the same α‐tubulin as shown in **A**. The data presented here are representative sets from three independent experiments.

**Figure 2:**
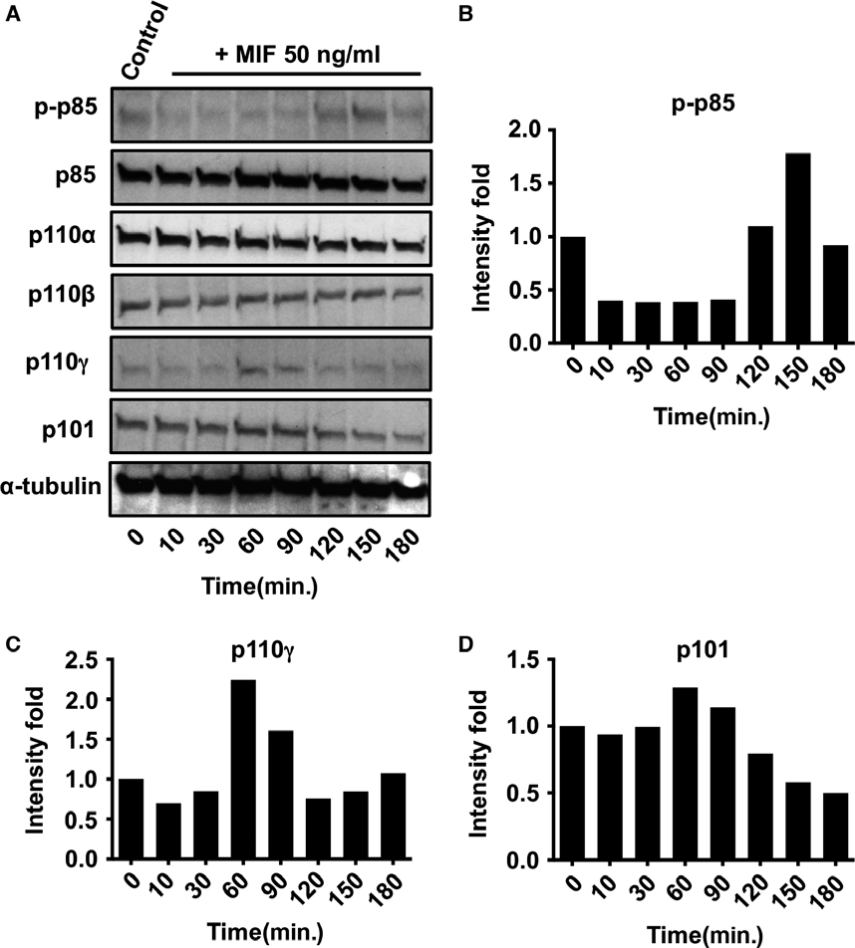
MIF activation of Class IB PI3Ks expression in HL60 neutrophil‐like cells. (**A**) Western blots show the expression of phosphorylated‐p85, total p‐85, p110α, p110β, p110γ and p101 monitored from 10 to 180 min. after the MIF activation of HL60 cells. All controls were measured at *t* = 0 without MIF activation. (**B**–**D**) Bar diagrams show fold change in intensity for p‐p85, p110γ and p101 respectively. The intensity data were analysed by ImageJ software and normalized to the same α‐tubulin as shown in **A**. The data presented here are representative sets from three independent experiments.

As different PI3K isoforms account for distinct functions, identifying which isoforms are activated by MIF will clarify the mechanisms underpinning PI3K‐related signalling networks and lay the groundwork for understanding their regulation. Currently, reliable antibodies are available only for the p85, p101, p110α, β and γ isoforms. Among those, anti‐phospho antibody is only available for p85. Therefore, we examined the changes in protein expression of each isoform and phosphorylation level of p85 in THP‐1 monocytes and HL60 neutrophil‐like cells after the treatment with 50 ng/ml of MIF. As MIF activation of extracellular signal‐regulated kinase 1/2 is biphasic (15 and 150 min.) 35, we measured the MIF activation of each PI3K isoform for 180 min. The early activation is because of the exogenous MIF and the late activation is because of newly synthesized MIF acting in an autocrine fashion. The expression levels of Class IA PI3Ks, p85, p110α and β (Fig. 1A) did not change within 180 min. in THP‐1 monocytes. However, the phosphorylation level of p85 peaked at 10 and 180 min. (Fig. 1A and B). In contrast, the expression of the Class IB catalytic subunit, p110γ peaked at 180 min. after MIF treatment (Fig. 1A and C). We also examined the expression level change in the Class IB regulatory subunit, p101, which up‐regulates the activity of p110γ, and it peaked at 30 and 120 min. (Fig. 1A and D). Because neutrophil is one of the most MIF‐responsive cell types, we repeated the above assays with HL60 neutrophil‐like cells to compare them to THP‐1 monocytes. Similar to THP‐1 cells, the expression levels of p85, p110α and β did not change (Fig. 2A). However, the late‐stage phosphorylation of p85 was faster (peaked at 150 min.) in HL60 cells than in THP‐1 cells (peaked at 180 min.) (Fig. 2A and B). For p110γ and p101, there was only one peak expression at 60 min. compared to THP‐1 cells (Fig. 2A, C and D). Together, these results suggest that pre‐existing p85 in THP‐1 monocytes gets phosphorylated in response to the exogenous MIF within 10 min. However, it remains unclear whether this early‐stage activation is required for the late‐stage activation. For Class IB PI3K isoforms though, early‐stage induction of protein expression seems not critical for the late‐stage induction. In HL60 neutrophils, there is no rapid phosphorylation of p85. Instead the induction of p101 and p110γ expression starts at 60 min. and the phosphorylation of p85 occurs at 150 min. Therefore, the activation of PI3K may (for monocytes) or may not (for neutrophils) be important for the induction of isoform expression.

### MIF active site inhibitors inhibit the expression of p110γ and p101 PI3K subunits

**Figure 3:**
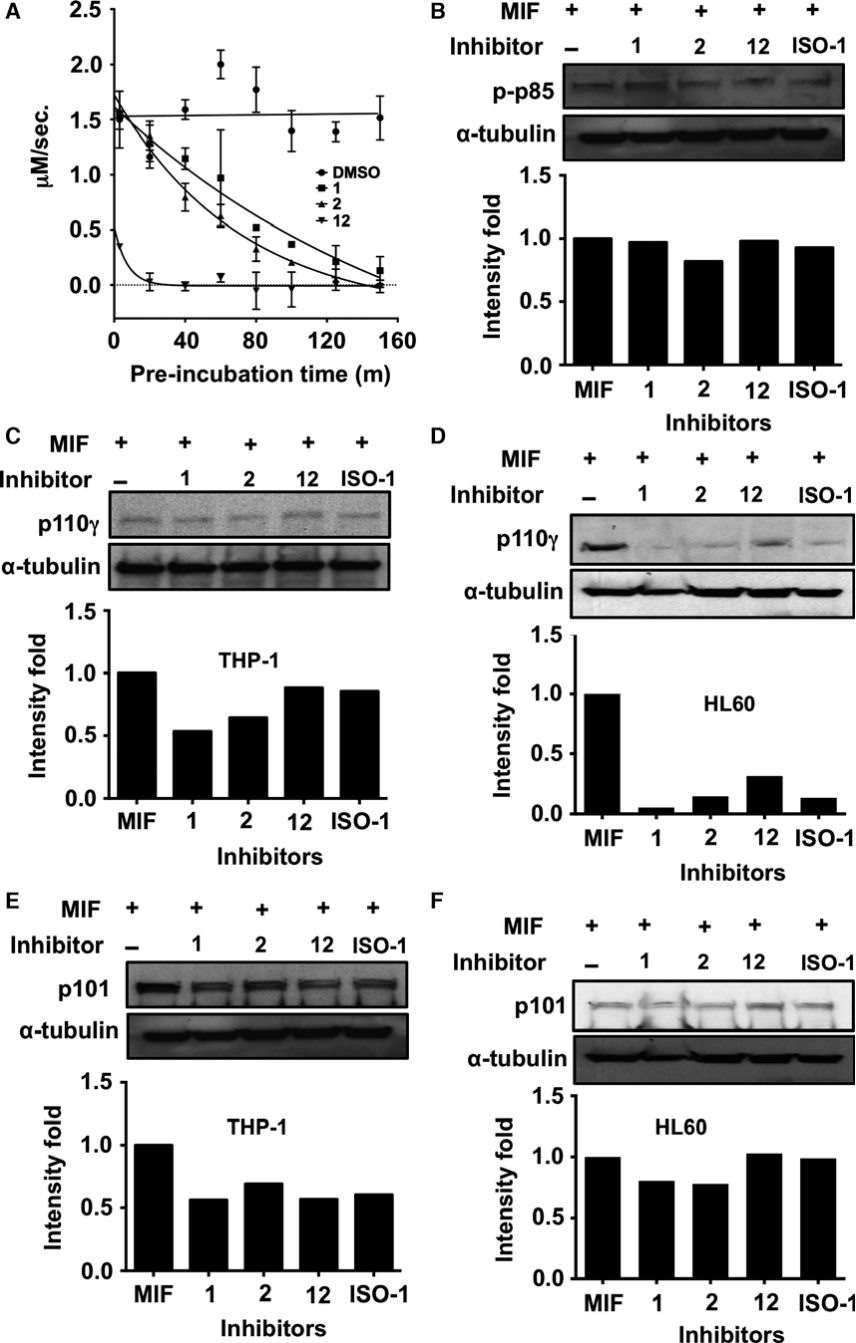
Inhibition of Class I PI3Ks expression by MIF active site inhibitors. (**A**) Each inhibitor was dissolved in DMSO and was incubated with MIF for different length of time prior to HPP tautomerase enzyme assay. As a negative control, DMSO alone was incubated with MIF. The enzyme activity data shown here are a representative set among three repeats with triplicates. (**B**) Phosphorylation status of p85 was examined in THP‐1 cells after 180 min. of pre‐incubation with MIF in the absence and presence of active site inhibitors. Reduction in p110γ (**C**) and p101 (**E**) subunits expression by MIF inhibitors in THP‐1 cells was examined. Reduction in p110γ (**D**) and p101 (**F**) subunits expression by MIF inhibitors in HL60 cells was examined.

We kinetically characterized three MIF inhibitors (**1**,** 2** and **12**) for use as molecular probes to identify the structural determinants on MIF associated with the activation of PI3Ks. **1** and **12** are new inhibitors and **2** was recently reported elsewhere, but numbered differently 26. We conducted time‐dependent HPP tautomerase inhibition assays as described 36 and confirmed covalent bonding of these inhibitors with MIF (Fig. 3A). Residual tautomerase activity of MIF declined with inhibitor pre‐incubation time. This is consistent with covalent bond formation between the inhibitors and MIF as confirmed by X‐ray crystallography in this study. The time required for **2** and **1** to inhibit 50% of MIF was 50 and 70 min. respectively. In contrast, **12** showed the complete covalent bond formation with MIF within 30 min. Furthermore, the extrapolated MIF activity in the presence of **12** at time 0 was 33%. This suggests that **12** may have both covalent and non‐covalent tautomerase inhibitory activity as described for a known covalent inhibitor, NAPQI 27. We then tested whether the phosphorylation of p85 in THP‐1 cells was affected by the binding of MIF active site inhibitors to MIF (Fig. 3B). Especially, we looked at the later phase activation level of p85 because it is much higher than the earlier phase and still reflects the inhibitory effect of MIF inhibitors. None of the active site inhibitors and a known competitive MIF inhibitor, ISO‐1 23, significantly reduced the MIF‐induced phosphorylation of p85 after 180 min. This implies that the phosphorylation of p85 may occur though an unknown receptor‐binding site on MIF, with which none of the inhibitors above can interfere. In contrast, p110γ expression was significantly down‐regulated by **1** and **2** in THP‐1 monocytes (Fig. 3C) and by all the tested inhibitors in HL60 neutrophil‐like cells (Fig. 3D). **12** exhibited relatively lower inhibition in both the cell types. As p101 plays an important role in amplifying the activity of p110γ 12, we tested whether the MIF inhibitors affect p101 expression. **2** inhibited the expression by 30% in THP‐1 cells, whereas other inhibits inhibited by around 45% (Fig. 3E). In contrast, the MIF inhibitor inhibition of p101 was lower in HL60 cells compared to THP‐1 cells. **1** and **2** inhibited by 25%, whereas **12** and ISO‐1 did not (Fig. 3F). We treated THP‐1 and HL60 cells with inhibitors alone and confirmed that there was no cytotoxicity (e.g. apoptotic and necrotic effects) (Fig. S1A and B). Most of the tested cells (>90%) were healthy and did not show any evidence of apoptosis. Our results demonstrate that binding of structurally diverse MIF active site inhibitors affect the expression of specific Class I PI3Ks in a cell‐type‐dependent manner.

### The expression levels of p110γ and p101 are correlated with the extent of leucocyte migration

**Figure 4:**
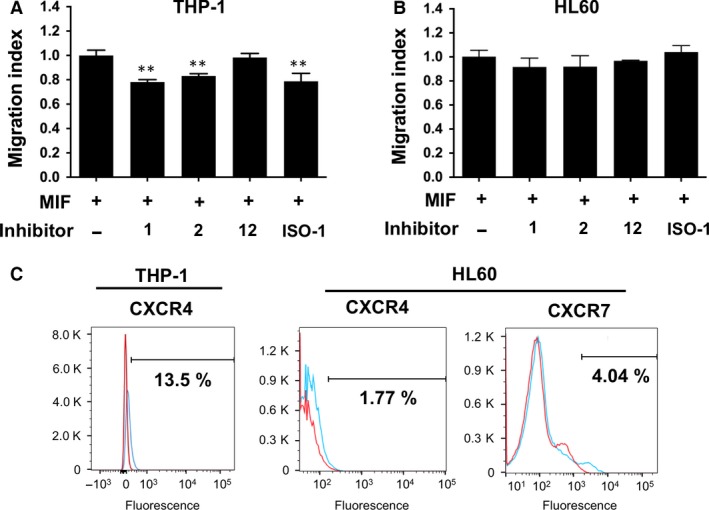
MIF‐induced chemotaxis inhibition assay. MIF‐induced migration of THP‐1 (**A**) and HL60 (**B**) cells was measured in the absence and presence of MIF active site inhibitors. A known MIF biological antagonist, ISO‐1, was used as a positive control. All the assays were carried out in triplicates. Statistical significance was analysed by unpaired Student's *t*‐test (**P < 0.005). (**C**) Expression of MIF receptors on the surface of THP‐1 and HL60 cells was analysed by flow cytometry. Isotype controls (red) and receptors (cyan) are coloured differently and overlaid for comparison.

To test whether the MIF inhibitor effect on p110γ and p101 expression is related to a biological activity of MIF, we conducted chemotaxis inhibition assay in the presence of the MIF inhibitors as described in the Experimental Procedures. Because the MIF‐induced PI3K expression is biphasic during the incubation time for chemotaxis assay, we correlate the later phase PI3K expression level and the end‐point cell migration data. **1**,** 2** and ISO‐1 significantly inhibited MIF‐induced THP‐1 migration (*P* = 0.005), whereas **12** did not (Fig. 4A). This is consistent with the relatively lower inhibitory effect of **12** on the expression of p110γ and p101 (Fig. 3C–F). In contrast, none of the inhibitors significantly inhibited the migration of HL60 cells (Fig. 4B). This suggests that HL60 chemotaxis may rely less on p110γ and p101 and may be compensated by other factors and signalling pathways. To find what MIF receptors were responsible for the expression of p110γ and p101, subsequent cell migration, and the inhibition by the MIF inhibitors, we performed flow cytometry to analyse the surface expression of known MIF receptors such as CXCR2, 4 and 7 and CD74. We observed only CXCR4 (13.5%) from THP‐1 cells while we observed CXCR4 (1.77%) and CXCR7 (4.04%) from HL60 cells (Fig. 4C). CD74 is thought to be responsible for lung neutrophil recruitment because the anti‐CD74 antibody abolishes the function 26. However, CD74 was not detected in the cell populations used here. The result was identical even after the activation of THP‐1 cells with IFN‐γ that was used to induce the expression of CD74 elsewhere 37. These results demonstrate that the expression levels of Class IB PI3Ks are related to the total number of MIF‐induced migrated THP‐1 monocytes. In addition, our flow cytometry analysis results are consistent with the previous report demonstrating that MIF uses not only CD74 but also chemokine receptors, CXCR2, CXCR4 and CXCR7 to induce the expression of Class IB PI3Ks 38. Involvement of G‐protein‐coupled chemokine receptors is also consistent with the G_βγ_ regulation of p101 and p110γ 39.

### Alteration of MIF surface by active site inhibitors does not fully explain the reduction in monocyte chemotaxis

**Figure 5:**
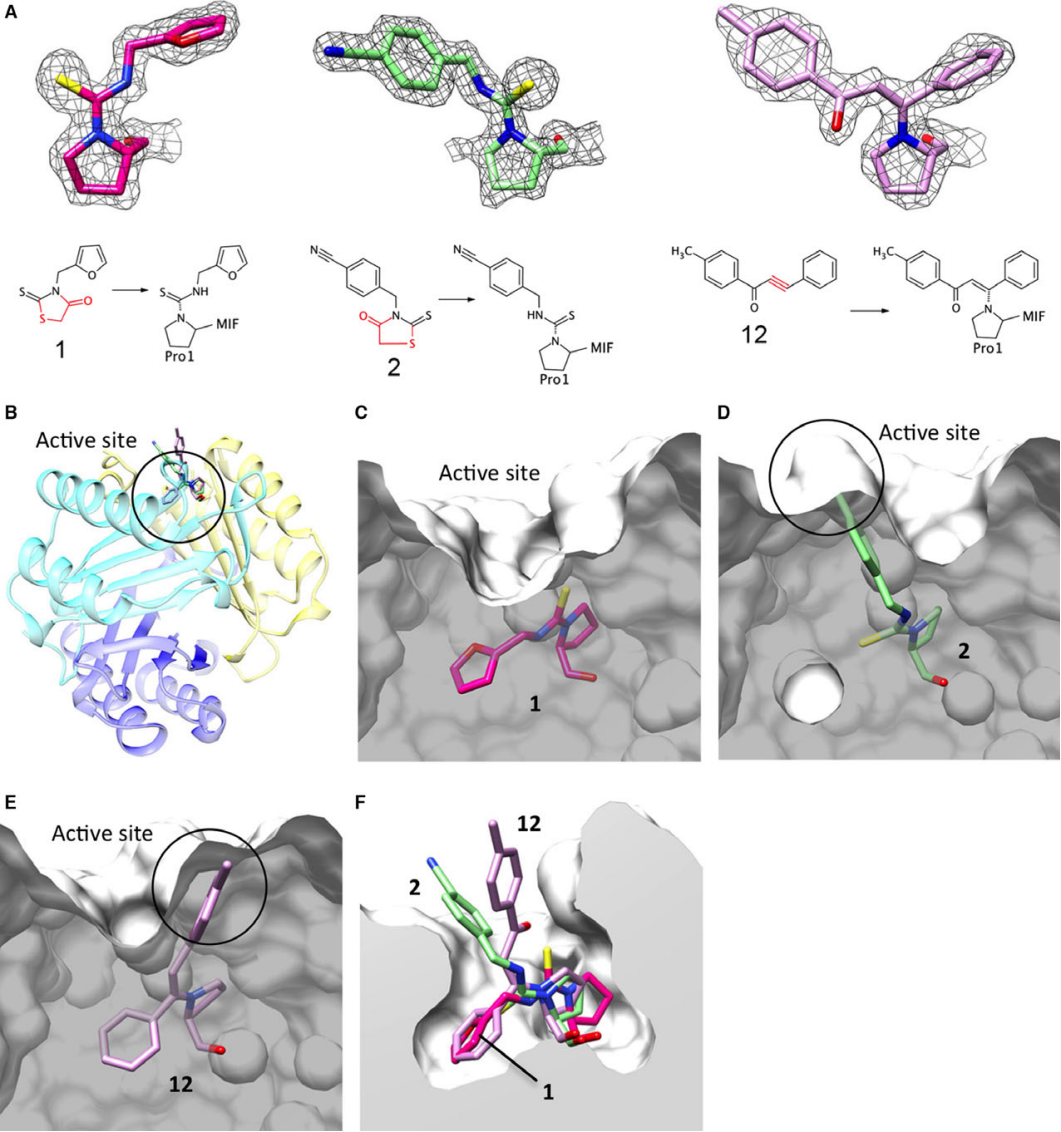
Alteration of the active site surface by MIF inhibitors. (**A**) Unbiased electron densities (F_o_‐F_c_) were generated without the structural models of the MIF inhibitors. The original and Pro1‐bound chemical structures of three covalent inhibitors are shown under the electron density. The leaving groups of the inhibitors are coloured in red. (**B**) Binding location of three inhibitors (**1**,** 2** and **12**) is shown on a MIF trimer. Each monomer is coloured differently. (**C**–**F**) Surface of the active site is cut to show the side view of the occupying inhibitors. Protein surface altered by each inhibitor is shown from a slightly different angle for **1** (**C**), **2** (**D**) and **12** (**E**). Modified surfaces by the solvent‐exposed rings of **2** and **12** are circled. (**F**) Inhibitors bound to the active site were superposed to compare their relative binding positions.

**Figure 6:**
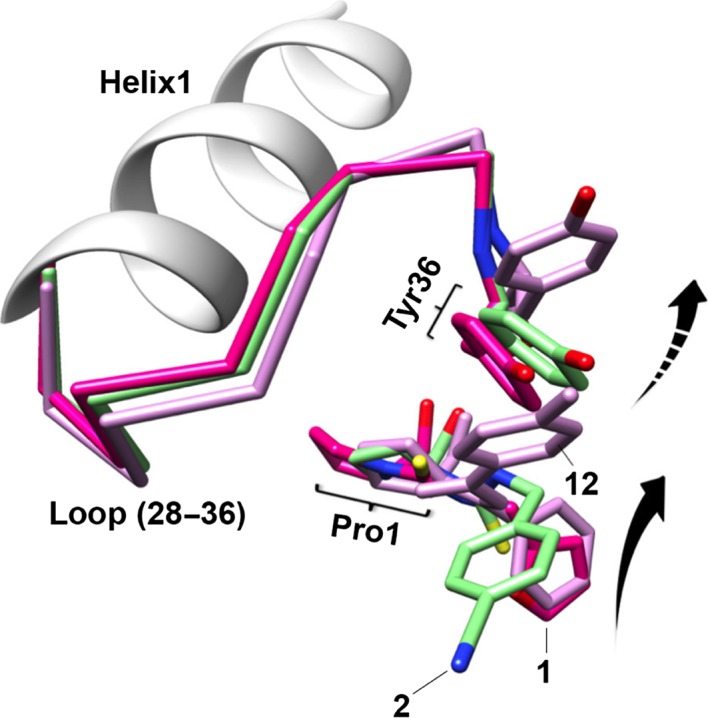
Structural changes of L2 and Tyr36 caused by MIF active site inhibitors. Superposition of the inhibitor‐bound MIF monomers shows the rotational shifts (dashed arrow) of Tyr36 away from Pro1 depending on the relative position of the solvent‐exposed ring of each inhibitor (solid arrow). Loop (28‐36), the L2 loop, shows structural flexibility upon inhibitor binding.

We previously proposed that the alteration of the active site‐solvent boundary by small molecule MIF inhibitors is related to the inhibition of MIF binding to receptors 26. To structurally verify the proposal for the inhibition of p110γ and p101 expression, we co‐crystallized **1**,** 2** and **12** with MIF. Structural models for the three covalent inhibitors were built into the electron densities based on the proposed covalent bond formation between the inhibitors and the first proline as shown under each inhibitor model (Fig. 5A). The crystallographic refinement statistics are summarized in Table S1. The leaving groups of the original inhibitors are coloured in red. Inhibitors bound to the MIF tautomerase active site formed between two neighbouring monomers within a MIF trimer are highlighted in stick representation (Fig. 5B). **1** occupies the bottom half of the catalytic pocket (Fig. 5C), whereas **2** occupies the upper half (Fig. 5D). **12** is covalently bound to Pro1 in the middle of its structure and as a result occupies more of the pocket compared to **1** and **2** (Fig. 5E). Because of this higher occupancy of the active site, **12** may stabilize better the conformation of the active site than **1** and **2**, resulting in no disruption of MIF interactions with its receptor. The solvent‐exposed ring structures of **2** and **12** point towards different directions (Fig. 5F). This suggests that the ring structure of **2** interferes the most with MIF receptor binding with the surrounding residues because **12** is less inhibitory than **2** against the biological activities of MIF (Figs 3 and 4). However, this steric hindrance theory does not explain the inhibition of MIF‐induced migration of THP‐1 cells and HL60 cells by **1** that does not have any solvent‐exposed ring structure like **2**. Thus, we superposed the crystal structures of the MIF:inhibitor complexes to investigate any structural changes on MIF upon inhibitor binding. The backbones of the apo‐ and inhibitor‐bound structures were well superposed (root‐mean‐square deviation in Å: 0.197 for **1**, 0.195 for **2**, 0.222 for **12** and 0.176 for ISO‐1) in most areas except the second loop (L2, residues 28–36) (Fig. 6). The only noticeable conformational change was found on the phenol ring of Tyr36. The phenol ring of Tyr36 was moved from the original position (magenta) as shown in **1**‐bound structure to a slightly open position (green) in **2**‐bound structure, and to the fully open position (light pink) in **12**‐bound structure. However, the position of the side chain of Tyr36 seems not significant for receptor binding because the MIF‐induced THP‐1 monocyte chemotaxis showed subtle or no effect with **12** (Fig. 3C–F). These results suggest that the alteration of the MIF surface by active site inhibitors is not the only case where MIF receptor interaction is affected, implying that probably the thermodynamic nature (or flexibility) of the receptor binding site on MIF needs to be investigated.

### Potent active site inhibitors increase the flexibility of the MIF L2 loop

**Figure 7:**
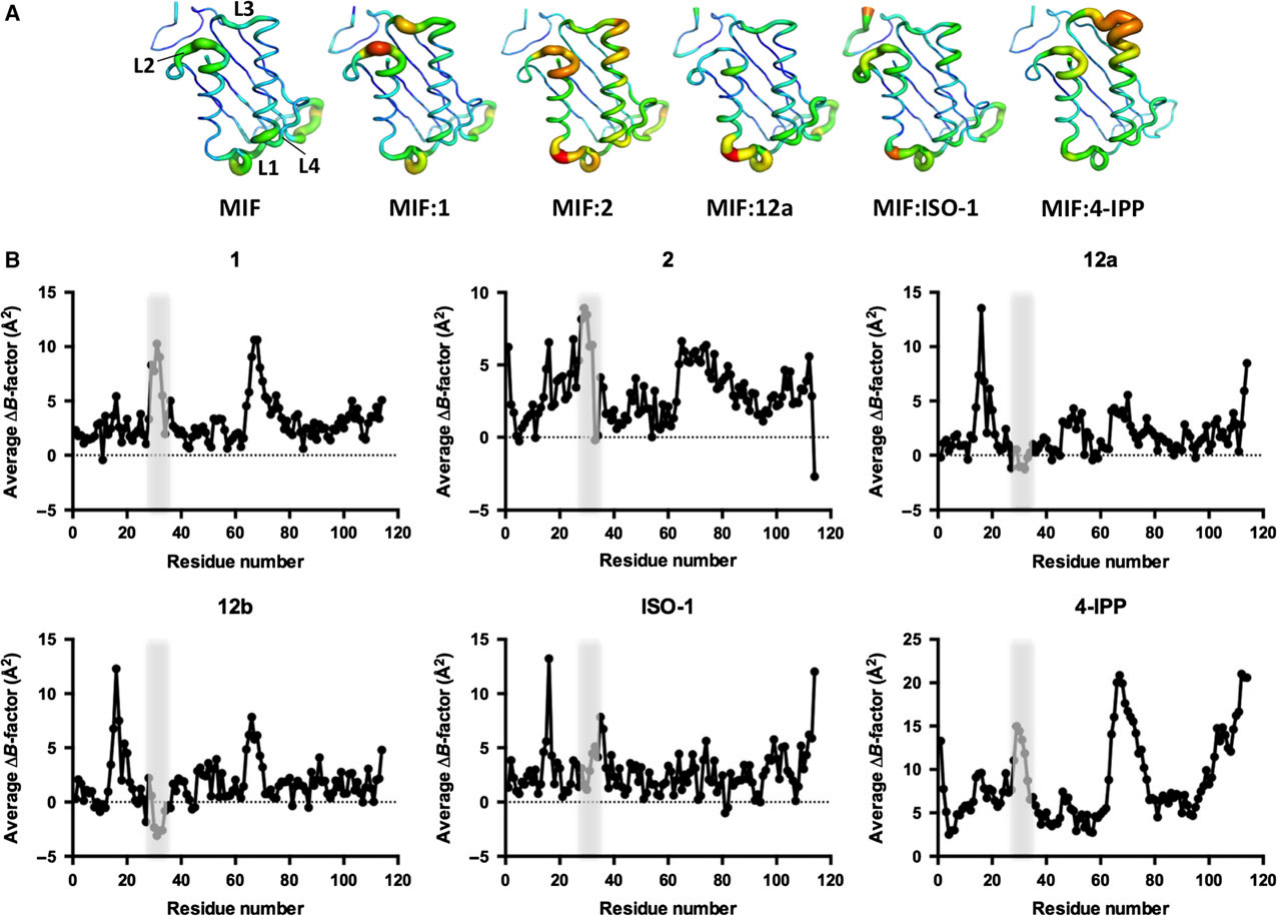
B‐factor analysis of MIF:inhibitor complex structures. (**A**) A monomer is presented for each MIF:inhibitor structure and loops are labelled (e.g. L1‐L4). B‐factor is represented in putty using PyMol from Schrödinger (New York, NY, USA). Low to high B‐factors are coloured in blue to red and drawn with narrow to wide tubing width. (**B**) Average B‐factor difference of a trimer per residue of each inhibitor‐bound MIF compared to MIF (CA atom only) is graphed. The residues belonging to the L2 loop are shaded.

We reported elsewhere that there is an inverse correlation between the flexibility of the active site and the extent of MIF‐induced neutrophil chemotaxis based on the structures of MIF active site mutants 26. To further analyse this relationship in the wild‐type MIF, we performed a B‐factor (or temperature factor) analysis for all the MIF:inhibitor complexes used in this study. The higher the B‐factor is, the more flexible the corresponding residue is. In crystal packing, MIF:**12** complexes revealed two trimers in a repeating three‐dimensional unit (or asymmetric unit), so each MIF trimer structure was analysed. As three monomers in each MIF trimer demonstrate very similar B‐factor profiles, one representative monomer for each MIF:inhibitor complex is shown in Figure 7A for easy comparison. The backbone of each MIF structure is depicted in B‐factor putty indicating high B‐factor in wider red tubing and low B‐factor in narrower dark blue tubing. To visualize how inhibitor binding affected individual residues, the average B‐factor difference (ΔB‐factor) between the apo‐MIF and each inhibitor‐bound MIF is shown in Figure 7B. All the structures exhibit similar B‐factors for L4 loop (see the first structure in Fig. 7A for the loop annotations). Apo‐MIF and MIF:**12** complexes exhibit B‐factors under 5 Å^2^ for L2 loop, indicating low flexibility and possibly stable interactions with receptors. In contrast, MIF:ISO‐1, MIF:**1** and MIF:**2** show relatively increased B‐factors (>10 Å^2^, >30 Å^2^, and >25 Å^2^ respectively) for the L2 loop. MIF:**1**, MIF:**2** and MIF:4‐iodo‐6‐phenylpyrimidine (4‐IPP) (PDB ID:3B9S) show high B‐factors for L3 loop, whereas MIF:ISO‐1 does not. This suggests that L3 loop is disturbed depending on inhibitors and may not be critical for receptor binding leading to PI3K expression. MIF:**12** shows high B‐factors only for L1 and this also suggests that L1 may not be critical for receptor binding. As MIF:**1**, MIF:**2** and MIF:ISO‐1 inhibited MIF‐induced leucocyte migration (Fig. 4) and increased B‐factors in L2, it is likely that structural stability of L2 is the critical structural determinant for the chemoattractant activity of MIF.

### The first proline displaces towards the MIF L2 loop upon inhibitor binding

**Figure 8:**
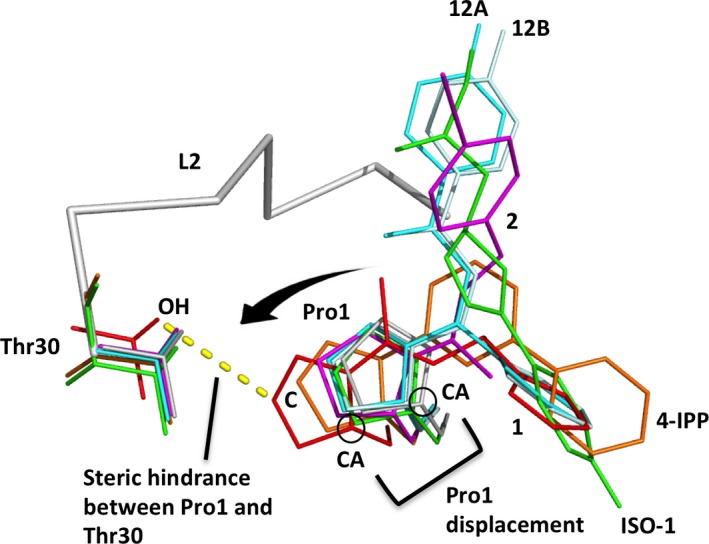
Displacement of inhibitor‐attached Pro1 towards the L2 loop. Pro1 residues with covalently attached inhibitors and corresponding Thr30 residues are depicted in sticks (Apo, light grey at the centre; ISO‐1, green; **12**, regular (A) and light (B) cyan; **2**, magenta; 4‐IPP, orange; **1**, red). The direction and the degree of the Pro1 displacement towards Thr30 are indicated with the arrow and its thickness, respectively.

To further understand the structural basis of the increased flexibility of L2, we scrutinized the positions of the inhibitor‐attached Pro1 relative to Thr30 belonging to L2 loop (Fig. 8). We included a known covalent MIF inhibitor, 4‐IPP 40 ‐bound MIF structure for a comparison with **1** because 4‐IPP demonstrated higher potency than ISO‐1 against cell migration and anchorage‐independent growth of lung cancer cells without any solvent‐exposing ring structure 40. The covalently attached Pro1 and 4‐IPP are three‐dimensionally aligned well with the covalently attached Pro1 and **1**. To examine how much the Pro1 located at the centre of the active site was displaced upon inhibition binding, which is important to understand the change in protein integrity and flexibility, we measured the distance between the CA of Pro1 covalently attached to each inhibitor and the CA of the apo‐MIF Pro1. Pro1 attached to **1** revealed the longest displacement (1.9 Å) followed by 4‐IPP (1.1 Å), **2** (0.8 Å), **12** (0.5 Å) and ISO‐1 (0.4 Å). This suggests that **1** might disturb the protein the most. Also, to examine steric hindrance between the inhibitor‐attached Pro1 and L2 loop, we measured displacement of the closest carbon of each Pro1 towards the side chain oxygen of Thr30. The displacements are 1.1 Å for **1**, 0.6 Å for 4‐IPP, 0.6 Å for **2** and 0.5 Å for ISO‐1 and 0.2 Å for **12**. This suggests that the displacement of Pro1 upon inhibitor binding within the tight active site pushes and destabilizes the L2 loop, resulting in suboptimal MIF binding to receptor.

### Neutrophil recruitment *in vivo* is inhibited by inhibitor 1 but not inhibitor 12

**Figure 9:**
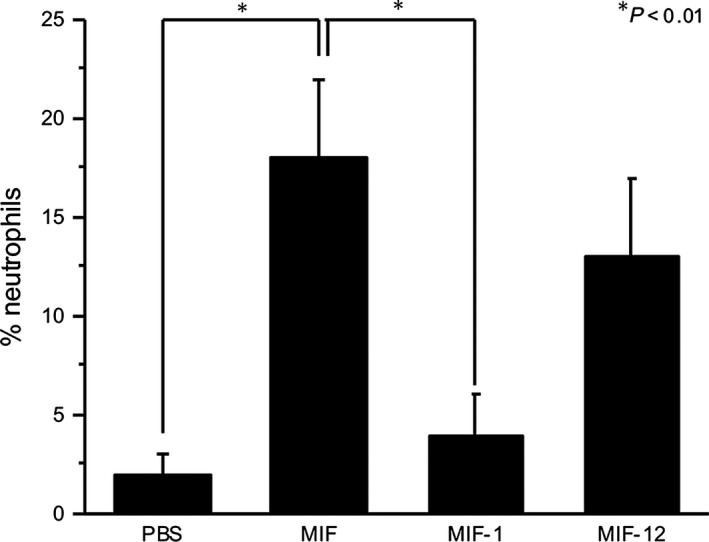
Lung neutrophil recruitment inhibition assay. The Y‐axis is the percentage of neutrophils among cells found in the bronchoalveolar lavage after MIF:inhibitor complex treatment. Reduction in neutrophil recruitment after treating with MIF‐1 (MIF pre‐incubated with **1**) is statistically significant compared to MIF alone (*P* < 0.01).

To verify the physiological consequences of the aforementioned structure‐function relationships, we treated mice with MIF alone or MIF:inhibitor complex and investigated how many neutrophils were inhibited to migrate by each inhibitor (Fig. 9). **1** inhibited 79% of the total number of neutrophils otherwise recruited by MIF. In contrast, **12** did not significantly inhibit neutrophil recruitment. **2** was not tested in this study as it revealed 57% of inhibition elsewhere 26. These results are consistent with those of the reduced expression of PI3K isoforms and the inhibited cell chemotaxis (Figs 3 and 4). This supports that our structural interpretation for the pharmacological inhibition of MIF‐induced PI3K signalling is physiologically valid.

## Discussion

We evaluated the expression levels of Class I PI3K catalytic subunits, p110α, β and γ, after MIF treatment of THP‐1 monocytes and HL60 neutrophil‐like cells. The expression level of p110γ increased, but the expression level of p110α and β did not change. Likewise, the expression of the regulatory subunit of p110γ increased in both the cell lines. The time required to reach to the later phase expression of p110γ was 90 min. earlier for HL60 cells when compared with THP1 cells. Neutrophils are the first responders to infection, so highly motile and rapidly congregate at the infection site and provide signals for the activation of macrophages 41. Monocytes are precursors to macrophages that live much longer than neutrophils and participate in antigen presentation. This motile and quickly responding characteristic of neutrophils may reflect the faster increase in the later phase expression of p110γ in HL60 cells compared to THP‐1 cells. This later phase expression of the PI3Ks is because of newly synthesized and secreted MIF. Thus, the autocrine MIF activation of neutrophils seems faster than monocytes. Another observation is that p101 and p110γ expression was peaked at 150 min. in THP‐1 cells and 60 min. in HL60 cells (Fig. 2C and D), which are 30 and 90 min., respectively, quicker than the phosphorylation of p85. This suggests that Class IB PI3Ks may be mainly responsible for the quick autocrine response to MIF in neutrophils and monocytes. Macrophage migration inhibitory factor has been shown to bind its surface receptors CD74, CXCR4 and CXCR2. Macrophage migration inhibitory factor binding to CD74 results in CD44‐dependent activation of Src‐family receptor kinases (RTK). Macrophage migration inhibitory factor can also bind and signal through G‐protein‐coupled chemokine receptors (CXCR2 and CXCR4) individually. Complex formation of CXCR2 with CD74 may facilitate GPCR activation and the formation of a GPCR‐RTK‐like signalling complex, leading to enhanced activation of PI3K. Blocking CXCR2 and CD74 antibodies was shown to impair MIF‐induced monocyte transmigration, providing further evidence for MIF‐induced leucocyte chemotaxis 6.

None of the inhibitors tested here inhibited the phosphorylation of p85 in THP‐1 monocyte cells (Fig. 3B) but did inhibit MIF‐induced chemotaxis in the same cell type (Fig. 4A). This suggests that monocyte chemotaxis is mainly driven by Class IB PI3Ks. In addition, our flow cytometry analysis revealed that THP‐1 cells had only CXCR4 on the surface, whereas HL60 cells had both CXCR4 and CXCR7. As the activation of CXCR7 leads to the activation of PI3K pathway 42, dual receptor could generate stronger signal than a single receptor system.

**12** increased the flexibility of L1, whereas other inhibitors did not. L1 contains Arg11 that is a part of the pseudo‐(E)LR C‐X‐C chemokine receptor binding motif 43. Thus, it is likely that **12** disrupts L1 interaction with either CXCR2 or CXCR4 and is also responsible for the partial inhibition of p110γ expression. ISO‐1 exhibited B‐factors (>10) between **1** (>30) and **12** (<5). However, it exhibited an equal level of inhibitory effect with **1** and **2** against the MIF‐induced THP‐1 cell migration. Thus, it suggests that ISO‐1 partially interferes receptor binding with the solvent‐exposed ring structure as **2** does with minimal disturbance of L2. In fact, the solvent‐exposed ring structure of ISO‐1 and **2** are well overlaid.

In summary, our study suggests that the L2 loop of MIF plays a pivotal role in activating the expression of Class IB PI3Ks, p101 regulatory subunit and p110γ catalytic subunit. We also demonstrate that structurally diverse MIF active site inhibitors can be used as molecular probes to elucidate the molecular basis of potential receptor binding sites on MIF and the activation of downstream signalling pathways.

## Accession numbers

The structural coordinates of MIF in complex with **1**,** 2** and **12** were deposited to the Protein Data Bank (PDB) and their PDB codes are 4Z15, 4Z1T, 4Z1U, respectively.

## Conflict of interest

Authors have no conflict of interest to declare.

## Supporting information


**Table S1** Crystallographic data collection and refinement statistics.
**Figure S1** PI/Annexin cell viability assays.
