# Supplementary material for: Structural basis for decreased induction of class IB PI3‐kinases expression by MIF inhibitors
Source: J Cell Mol Med. 2016 Sep 13;21(1):142–53. doi: 10.1111/jcmm.12949 (PMC5192866; doi:10.1111/jcmm.12949)
Supplement: Supplementary file 1 — Table S1 Crystallographic data collection and refinement statistics. Figure S1 PI/Annexin cell viability assays. [file JCMM-21-142-s001.doc]

Table S1. Crystallographic data collection and refinement statistics.

|  | MIF:1 | MIF:2 | MIF:12 |
| --- | --- | --- | --- |
| **Data collection** |  |  |  |
| Space group | P2_1_2_1_2_1_ | P2_1_2_1_2_1_ | P2_1_2_1_2_1_ |
| Cell dimensions |  |  |  |
| a, b, c (Å) | 67.7, 68.1, 88.0 | 68.1, 68.6, 88.2 | 94.9, 105.9, 106.7 |
| *(Alpha),(beta),(gamma)* (°) | 90, 90, 90 | 90, 90, 90 | 90, 90, 90 |
| Resolution (Å) | 1.6 | 1.5 | 2.1 |
| *R_sym_ or R_merge_^a^* | 0.062 | 0.067 | 0.101 |
| *I /(sigma)I* | 6.2 | 5.0 | 6.0 |
| Completeness (%) | 98.8 | 98.9 | 99.9 |
| Redundancy | 6.4 | 4.6 | 6.9 |
|  |  |  |  |
| **Refinement** |  |  |  |
| Resolution (Å) | 1.6 | 1.5 | 2.1 |
| No. reflections | 54,348 | 66,892 | 64,645 |
| *R_work_^c^ / R_free_^d^* | 0.198/0.228 | 0.202/0.229 | 0.219/0.245 |
| No. atoms |  |  |  |
| Protein | 2,498 | 2,496 | 5,020 |
| Ligand/ion | 48 | 48 | 154 |
| Water | 151 | 185 | 267 |
| B-factors |  |  |  |
| Protein | 19.3 | 22.5 | 18.7 |
| Ligand/ion | 19.5 | 23.4 | 14.4 |
| Water | 25.6 | 21.6 | 27.5 |
| R.m.s deviations |  |  |  |
| Bond lengths (Å) | 0.024 | 0.029 | 0.020 |
| Bond angles (°) | 2.245 | 2.585 | 2.112 |

^a^R_merge_ = $\left(\sum\left|I_{i}-\left\langle I_{i}\right\rangle\right|\right)/\sum\left|I_{i}\right|$, where $\left\langle I_{i}\right\rangle$ is the mean intensity over all symmetry-equivalent reflections.

^b^R_work_ = $\left(\sum\left|\left|F_{o}\right|-\left|F_{c}\right|\right|\right)/\sum\left|F_{o}\right|$, where $F_{o}$ and $F_{c}$ stand for observed and calculated structure factors, respectively.

^c^Five percent of the entire reflection were used for calculation of R_free_.

Single data set collected from a single crystal was used for each structure.

There was one MIF molecule in asymmetric unit for MIF:1 and MIF:2 complexes and were two MIF molecules for MIF:12 complex.


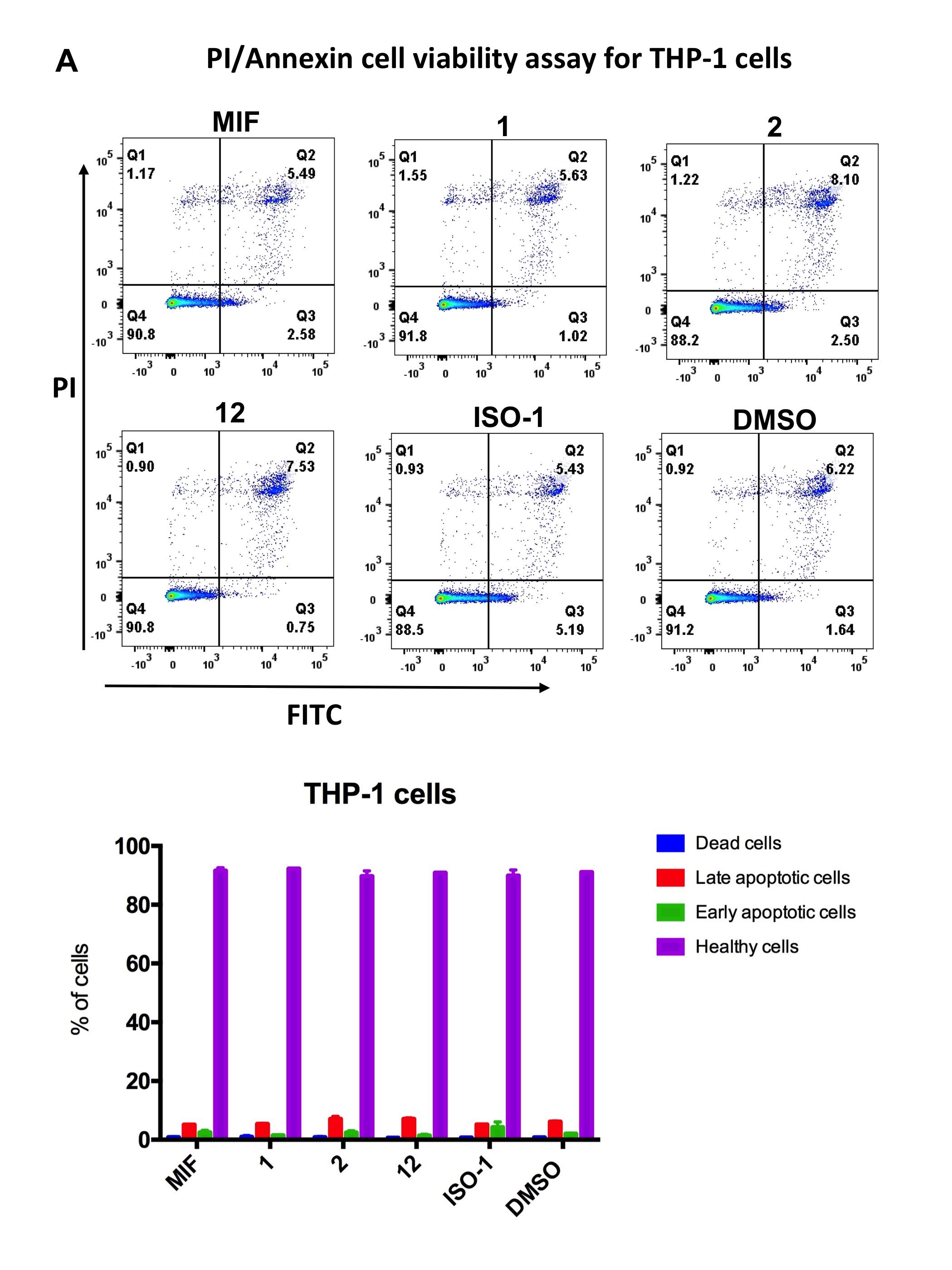


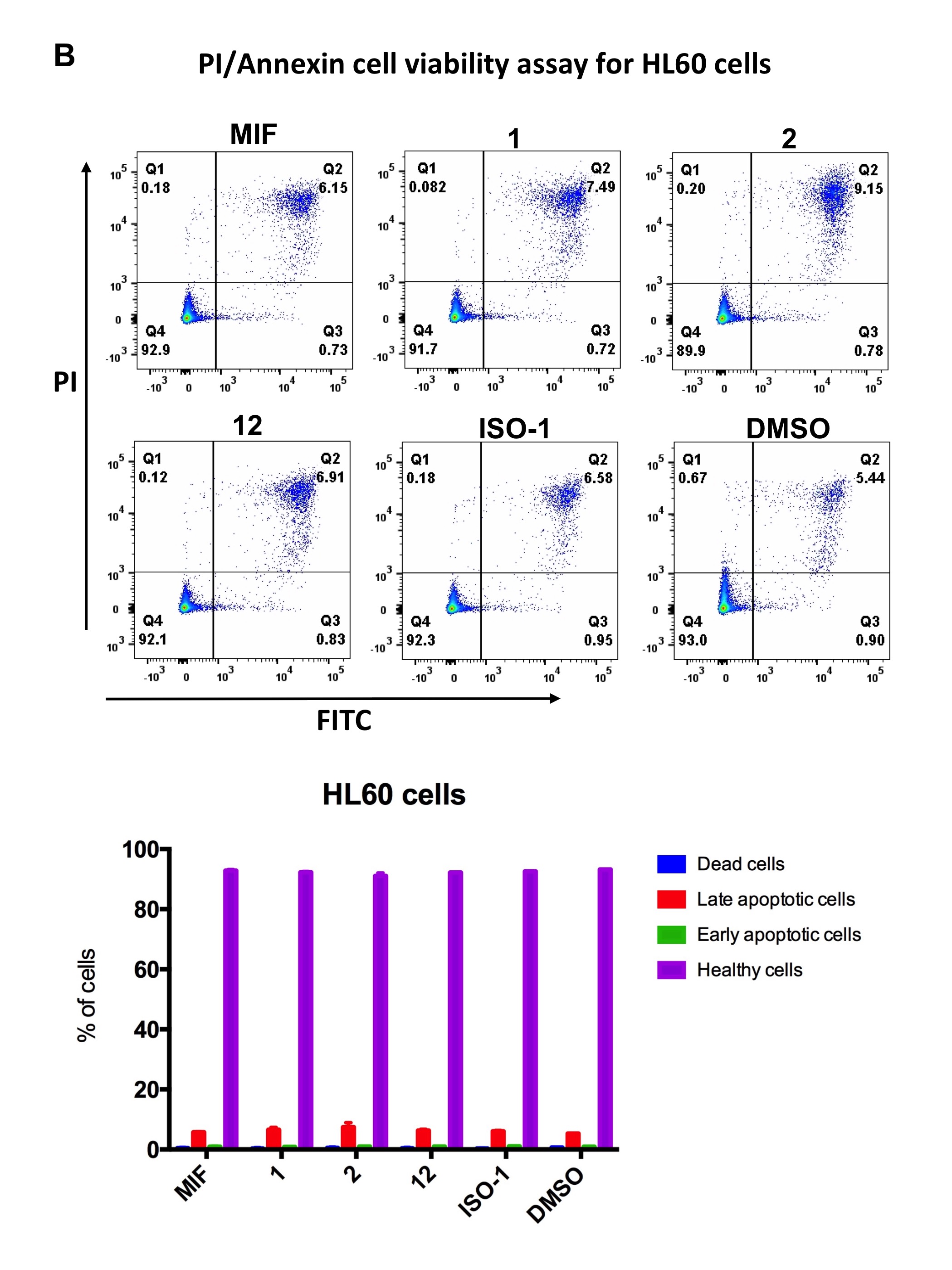


Supplementary figure 1. PI/Annexin cell viability assays.

THP-1 monocytes (A) and HL60 neutrophil-like cells (B) were treated with MIF or MIF active-site inhibitors for 180 min and stained with Annexin-V-FITC and PI. We repeated this twice. A representative dot plot of FITC vs. PI per each inhibitor and MIF is presented. Corresponding bar diagrams are drawn with mean ± standard deviation for percentage healthy, early apoptotic, late apoptotic and dead cells. None of the inhibitors induced cell death in both the cell lines.
